# Ebola Virus Disease in Health Care Workers — Sierra Leone, 2014

**Published:** 2014-12-12

**Authors:** Peter H. Kilmarx, Kevin R. Clarke, Patricia M. Dietz, Mary J. Hamel, Farah Husain, Jevon D. McFadden, Benjamin J. Park, David E. Sugerman, Joseph S. Bresee, Jonathan Mermin, James McAuley, Amara Jambai

**Affiliations:** 1 Sierra Leone Ebola Response Team, CDC; 2 Division of Global HIV/AIDS, Center for Global Health, CDC; 3 CDC Zimbabwe; 4 National Center for HIV/AIDS, Viral Hepatitis, STD, and TB Prevention, CDC; 5 Division of Parasitic Diseases and Malaria, Center for Global Health, CDC; 6 Division of Global Health Protection, Center for Global Health, CDC; 7 Division of State and Local Readiness, Office of Public Health Preparedness and Response, CDC; 8 Division of Healthcare Quality Promotion, National Center for Emerging and Zoonotic Infectious Diseases, CDC; 9 National Center for Immunization and Respiratory Diseases, CDC; 10 CDC Zambia; 11 Ministry of Health and Sanitation, Sierra Leone

Health care workers (HCWs) are at increased risk for infection in outbreaks of Ebola virus disease (Ebola) (1). To characterize Ebola in HCWs in Sierra Leone and guide prevention efforts, surveillance data from the national Viral Hemorrhagic Fever database were analyzed. In addition, site visits and interviews with HCWs and health facility administrators were conducted. As of October 31, 2014, a total of 199 (5.2%) of the total of 3,854 laboratory-confirmed Ebola cases reported from Sierra Leone were in HCWs, representing a much higher estimated cumulative incidence of confirmed Ebola in HCWs than in non-HCWs, based on national data on the number of HCW. The peak number of confirmed Ebola cases in HCWs was reported in August (65 cases), and the highest number and percentage of confirmed Ebola cases in HCWs was in Kenema District (65 cases, 12.9% of cases in Kenema), mostly from Kenema General Hospital. Confirmed Ebola cases in HCWs continued to be reported through October and were from 12 of 14 districts in Sierra Leone. A broad range of challenges were reported in implementing infection prevention and control measures. In response, the Ministry of Health and Sanitation and partners are developing standard operating procedures for multiple aspects of infection prevention, including patient isolation and safe burials; recruiting and training staff in infection prevention and control; procuring needed commodities and equipment, including personal protective equipment and vehicles for safe transport of Ebola patients and corpses; renovating and constructing Ebola care facilities designed to reduce risk for nosocomial transmission; monitoring and evaluating infection prevention and control practices; and investigating new cases of Ebola in HCWs as sentinel public health events to identify and address ongoing prevention failures.

For this report of Ebola in HCWs in Sierra Leone, data were analyzed on laboratory-confirmed cases in the national Viral Hemorrhagic Fever database, which was created to capture and analyze data from the 2014 Ebola outbreak. Surveillance officers used a standardized case investigation form to collect information from patients with suspected or probable Ebola (2) and their family members. Information collected included age, sex, address, occupation, date of onset of symptoms, and potential exposures to other Ebola patients. “Health care worker” was one of the choices listed under a patient’s occupation and included clinicians such as doctors and nurses, as well as members of other cadres, including ambulance drivers, hospital cleaners, and burial team members. Vital status and laboratory information were entered into the patient’s case record as results were reported to the surveillance team in each health district. District data were merged at the national level. Whole blood from live patients and oral swab specimens from corpses were sent to one of several laboratories in Sierra Leone. Reverse transcription–polymerase chain reaction assays were used to confirm *Ebolavirus* infection. Select characteristics of HCW and non-HCW cases were compared using chi-square tests. P-values <0.05 were considered significant. To inform infection prevention and control efforts and surveillance of Ebola in HCWs, unstructured interviews concerning HCW infections were conducted with HCWs and health facility administrators in the course of site visits to health care facilities in eight districts during August–October 2014.

During May 23 through October 31, 2014, there were 3,854 laboratory-confirmed cases of Ebola reported in Sierra Leone in the Viral Hemorrhagic Fever database, including 199 cases in HCWs (5.2%). Seven additional cases in HCWs and 949 cases in non-HCWs had dates of symptom onset that were missing or outside of May 23 (date of the first documented case) to October 31 and were excluded from analysis. According to the *National Health Strategic Plan 2010–2015*, published in 2009 (3), Sierra Leone had a total health workforce of 2,402 persons. Using this denominator, the cumulative confirmed Ebola incidence in HCWs was 8,285 per 100,000. This can be compared with the 2,806 confirmed Ebola cases in non-HCWs in a national population of 3.49 million persons aged ≥15 years, with a cumulative incidence in adult non-HCWs of 80.4 per 100,000 population. Therefore, the confirmed Ebola incidence was 103-fold higher in HCWs than that in the general population in Sierra Leone.

Among confirmed cases in HCWs, 54.8% were in males, compared with 48.2% in non-HCWs (p=0.09). Of 183 (92%) confirmed Ebola cases in HCWs with recorded age, two (1.1%) were reportedly in persons aged <15 years, 82.0% were in persons aged 15–49 years, and 16.9% were in persons aged ≥50 years. There were no confirmed Ebola cases in HCWs reported in May. The number peaked at 65 cases in August and declined to 36 in September and 42 in October (Figure 1). The highest percentage of confirmed Ebola patients that were HCWs was in August (9.2%); this declined to 3.5% in October (Figure 1). The number of confirmed Ebola cases in HCW per district ranged from zero in two districts to 65 cases in Kenema District (Figure 2), which also had the highest percentage of all confirmed Ebola patients that were HCWs (12.9%). District of residence was missing in seven cases in HCWs (3.5%).

The surveillance form included questions on potential sources of infection, specifically attendance at a funeral or contact with a person with known or suspected Ebola, with an ill person, or with a corpse in the month before onset of symptoms. Among 159 (80%) confirmed HCW Ebola cases with data on funeral attendance, 13.8% had attended a funeral, compared with 32.3% in non-HCW (p <0.001). Data on contact with a known or suspected Ebola patient or ill person or a corpse was available for 143 (72%) confirmed HCW Ebola cases; 18.2% were in persons who had contact with a person with known or suspected Ebola or an ill person, compared with 12.3% in non-HCWs (p = 0.05); 30.1% had contact with a corpse, compared with 34.3% in non-HCWs (p=0.3).

Among confirmed HCW Ebola patients, 12.1% were dead at the time of surveillance recording, compared with 15.0% among non-HCW patients (p=0.3); other data on vital status, including numbers with missing data at time of surveillance recording and final outcome, are not consistently available in the Viral Hemorrhagic Fever data.

Site visits and unstructured interviews with HCWs and health facility administrators revealed a broad range of circumstances potentially leading to Ebola in HCWs. These included a lack of standard operating procedures and clearly assigned responsibilities for infection prevention and control; overall staff shortages and lack of infection prevention specialists; limited availability of safe transport vehicles for patients and corpses; incorrect triage or recognition of potential Ebola in patients and corpses, including no reassessment of admitted patients to identify new symptoms of Ebola (especially children aged <5 years); delayed laboratory diagnosis of Ebola cases because of long turn-around time for specimen transport and reporting of results; inadequate control of Ebola patient or HCW movement within health facilities; and lack of delineation between high-risk and low-risk Ebola zones. Other findings included limited availability of appropriate personal protective equipment and hand washing facilities, including lack of water and sufficient chlorine supplies; no or inadequate training about and monitoring of personal protective equipment use and hand washing; lack of equipment and materials and no or inadequate training about and monitoring of decontamination of transport vehicles and care facility spaces; limited capacity and no or inadequate training about safe management of contaminated waste; and limited capacity and no or inadequate training about safe management and burial of corpses.

## Discussion

Analysis of the national Viral Hemorrhagic Fever database found 199 cases of Ebola in the Sierra Leone health workforce. Using the number of HCWs reported in 2009 (3) as a denominator for HCWs and comparing with infection rates in the general population aged ≥15 years, the estimated confirmed Ebola incidence rate was approximately 100-fold higher in HCWs than in non-HCW adults in Sierra Leone.

The number and proportion of all confirmed Ebola patients that were HCWs peaked in August. The subsequent reductions might be attributable to concurrent implementation of infection prevention and control measures, including training and availability of personal protective equipment, and could reflect a closure of many health facilities and reduction in availability of health care services and HCW exposure as the outbreak progressed. However, many Ebola cases in HCWs continued to be reported in October. The highest number of confirmed Ebola cases and the proportion of all confirmed Ebola case that were HCWs occurred in Kenema District. There were 43 Ebola cases in HCWs in Kenema District in July and August, mostly among Kenema General Hospital staff. Inquiries about breaches of infection prevention and control at Kenema General Hospital indicated, among other problems, challenges with overall site management and administrative controls, such as correct and consistent triage and isolation of Ebola patients. Although some districts, such as Kenema, were more heavily affected, confirmed Ebola cases in HCWs have been reported in 12 of 14 districts in Sierra Leone, including all districts that have reported more than 35 confirmed Ebola cases. Also, although most cases in HCWs occurred in facilities operated by the Ministry of Health and Sanitation, including both general care facilities and those designated for Ebola care, there were a small number of confirmed Ebola cases in HCWs at Ebola care facilities established and managed by international implementing partners. These findings underscore the widespread challenges with infection prevention and control in Sierra Leone.

Compared with non-HCW patients, HCW patients were less likely to have attended a funeral and were more likely to have had contact with a live Ebola patient or ill person in the 30 days before symptom onset. However, a substantial proportion of both HCW and non-HCW Ebola patients reported funeral attendance or contact with a corpse, highlighting the overall importance of transmission from corpses in this outbreak. HCW patients were not significantly less likely than non-HCW patients to be dead at the time their cases were recorded by the surveillance system. The finding that 12% of HCW patients were dead at the time of recording indicates shortcomings in contact tracing, early case identification, and access to medical care, even in HCWs, who might have been expected to have better awareness and access to health care.

The findings in this report are subject to at least four limitations. First, public health surveillance data were incomplete, especially in the context of a health emergency in a resource-poor setting. It has been estimated that overall case numbers represent only one third to one half of all cases (4). Second, data on key information such as occupation was missing or might have been incorrect on many case investigation forms, and many cases were not included in the analysis because of missing or out-of-range dates of onset of symptoms. Third, members of some cadres, such as ambulance drivers, burial team members, and community health workers, might not have been consistently recorded as HCWs on case investigation forms or in the Ministry of Health and Sanitation 2009 report on the health workforce (3), and the number of health workers might have changed since 2009. As a result, these findings likely undercount the number of *Ebolavirus*-infected HCWs in Sierra Leone. However, Ebola reporting might be more complete for HCWs than non-HCWs, so the ratio of the Ebola cumulative incidence in HCWs compared with non-HCWs might be an overestimate. Finally, data on exposures are also likely to be incomplete. For example, the finding that contact with an Ebola patient or ill person was reported for only 19% of HCWs with Ebola is likely an underestimate.

A broad range of potential problems with infection prevention and control were reported at both general care facilities and those designated for Ebola care. The Ministry of Health and Sanitation, together with Sierra Leonean and international partners, are implementing a wide range of interventions, including policies, training, procurement, renovation, construction, and monitoring and evaluation, in accordance with established recommendations (5). As is the case with prevention of nosocomial transmission of tuberculosis (6), many observed breaches of infection prevention and control practices appeared to be attributed to failures of administrative controls, such as incorrect triage, or infrastructure limitations of renovated facilities, such as lack of barriers separating Ebola wards, rather than personal protective equipment failures; particular attention to these issues is recommended in the control of Ebola.

Cases of Ebola in HCWs are currently being investigated as sentinel public health events. An infection in an HCW might represent transmission from an Ebola patient in a health care facility, but might also be a signal for transmission to and from HCWs in the community, and for facility-based transmission from patient to patient and from HCWs to patients or to other HCWs. New, high-quality, dedicated Ebola treatment units are being established by international partners in Sierra Leone, but because the number of these beds does not meet the need in high-transmission areas, other, less well-resourced facilities, including Ebola care, holding, and isolation centers, are being established by the Ministry of Health and Sanitation. Given the high risk of nosocomial transmission of *Ebolavirus* (5), health authorities must be vigilant in implementation of strict infection prevention and control measures in all health care settings and alert to the possibility that less well-controlled settings might inadvertently act to propagate rather than interrupt transmission. Prevention of Ebola in HCWs is also critical to sustain the health workforce to address all causes of morbidity and mortality in Sierra Leone.

What is already known on this topic?

Health care workers (HCWs) are at increased risk for infection in outbreaks of Ebola virus disease (Ebola). Adherence to good infection prevention and control practices are required to prevent Ebola in HCWs.

What is added by this report?

As of October 31, 2014, of the total of 3,854 laboratory-confirmed Ebola cases reported from Sierra Leone, 199 (5.2%) were in HCWs. This was estimated to be a much higher cumulative incidence of confirmed Ebola in HCWs compared with non-HCWs. A broad range of breaches of good infection prevention and control practices were reported, and Ebola cases in HCW continued to be reported in October.

What are the implications for public health practice?

In Ebola outbreaks, comprehensive programs to reduce the risk for Ebola in HCWs in all health care settings are needed, including development of standard operating procedures (including safe triage), recruiting and training staff, procuring needed commodities and equipment, renovating and constructing safe Ebola care facilities, monitoring and evaluating infection prevention and control practices; and investigating new cases of Ebola in HCWs as sentinel public health events to identify and address ongoing prevention failures.

## Figures and Tables

**FIGURE 1:**
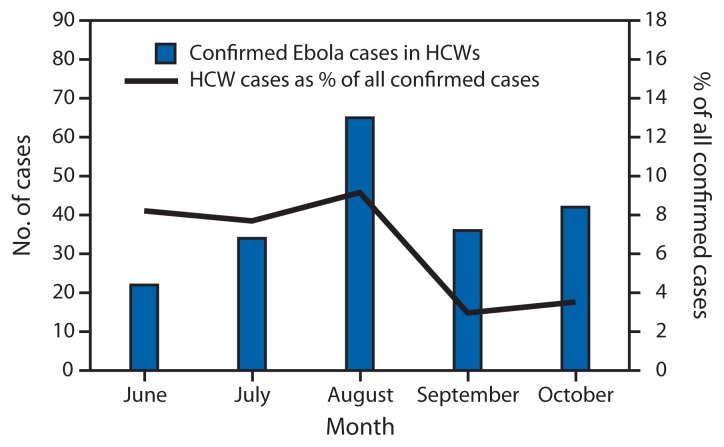
Number of laboratory-confirmed Ebola virus disease (Ebola) cases in health care workers (HCWs) and confirmed Ebola cases in HCWs as a percentage of all confirmed cases, by month — Sierra Leone, June–October 2014

**FIGURE 2:**
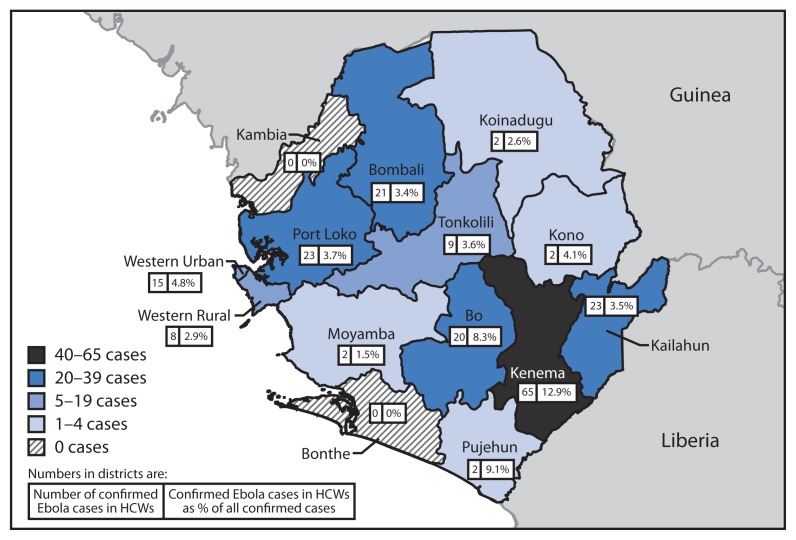
Number of laboratory-confirmed Ebola virus disease (Ebola) cases in health care workers (HCWs) and confirmed Ebola cases in HCWs as a percentage of all confirmed cases, by district — Sierra Leone, May–October 2014
